# Febrile neutropenia with bacterial paronychia

**DOI:** 10.1002/ccr3.1399

**Published:** 2018-01-26

**Authors:** Yuki Hosono, Takeshi Uenami, Yukihiro Yano, Masahide Mori

**Affiliations:** ^1^ Department of Thoracic Oncology National Hospital Organization Toneyama National Hospital Toyonaka Osaka 560‐8552 Japan

**Keywords:** Bacterial paronychia, chemotherapy, docetaxel, febrile neutropenia, lung cancer, onycholysis

## Abstract

The symptoms of infection can be minimal or absent in patients with febrile neutropenia at first. The focal site of infection, which may be the main cause of a fever or be a complication of neutropenia, can develop as neutrophils increase during the clinical course of febrile neutropenia.

A 69‐year‐old man presented to our hospital with fever (40.2°C). He had received eight courses of chemotherapy consisting of docetaxel and ramucirumab for nonsmall cell lung cancer 9 days prior to his admission and suffered from onycholysis as the side effect of docetaxel. His neutrophil count was 0.08 × 10^3^/μL. A physical examination including his skin revealed no abnormalities. He was diagnosed with febrile neutropenia with unknown origin. Four days after the administration of tazobactam/piperacillin and filgrastim, a swollen abscess with pain appeared around his fifth left fingernail (Fig. 1A). The neutrophil count increased to 4.24 × 10^3^/μL. We considered that the increased neutrophils had led to the development of the symptoms of bacterial paronychia, which might have caused fever or been a complication of neutropenia. Incision and drainage of the abscess resulted in defervescence (Figs. 1B and 2). *Streptococcus agalactiae* (Group B) and *Klebsiella oxytoca* were isolated from the abscess culture.

**Figure 1:**
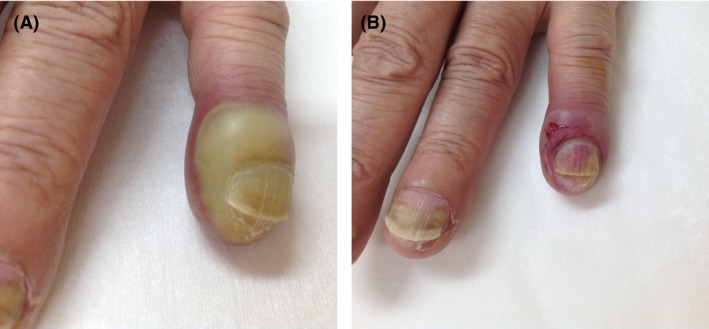
(A) Photograph of a swollen abscess around the patient's fifth left fingernail. (B) The fifth left finger after incision and drainage of the abscess.

**Figure 2:**
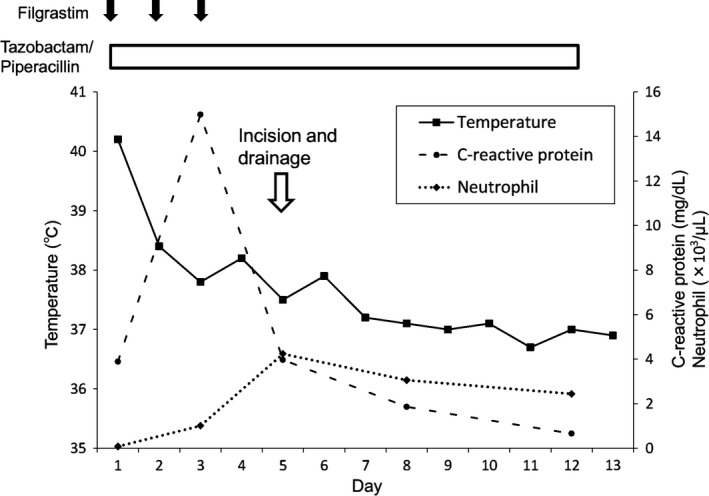
Clinical course of the patient.

Nail abnormalities during docetaxel treatment tend to be the result of bacterial infection, which can induce sepsis, as neutropenia often occurs during taxane therapy 1. This case emphasizes the importance of careful observation of febrile neutropenia patients, because the symptoms of infection can be minimal or absent at first 2.

## Conflict of Interest

None declared.

## Authorship

YH: procured the images and drafted the article. TU and YY: had advisory roles in the management of the patient. MM: reviewed and revised the manuscript.

## References

[1] Minisini, A. M. , A. Tosti , A. F. Sobrero , M. Mansutti , B. M. Piraccini , C. Sacco , et al. 2003 Taxane‐induced nail changes: incidence, clinical presentation and outcome. Ann. Oncol. 14:333–337. 12562663 10.1093/annonc/mdg050

[2] de Naurois, J. , I. Novitzky‐Basso , M. J. Gill , F. M. Marti , M. H. Cullen , and F. Roila . 2010 Management of febrile neutropenia: ESMO Clinical Practice Guidelines. Ann. Oncol. 21:252–256. 10.1093/annonc/mdq196 20555092

